# Association of dietary total antioxidant capacity with general and abdominal obesity in type 2 diabetes mellitus patients

**DOI:** 10.1371/journal.pone.0306038

**Published:** 2024-06-26

**Authors:** Najia El Frakchi, Khaoula El Kinany, Marwa El Baldi, Younes Saoud, Karima El Rhazi

**Affiliations:** 1 Laboratory of Applied Biology and Pathology (UAE/U24FS), Faculty of Sciences, Abdelmalek Essaadi University, Tetouan, Morocco; 2 Laboratory of Epidemiology and Research in Health Sciences, Faculty of Medicine, Pharmacy and Dental Medicine, Sidi Mohammed Ben Abdellah University, Fez, Morocco; Universidade de Tras-os-Montes e Alto Douro Escola de Ciencias da Vida e do Ambiente, PORTUGAL

## Abstract

**Background:**

The dual existence of Type 2 Diabetes Mellitus (T2DM) and obesity within a single individual may describe a combined adverse health effects, including impaired quality of life and increased risk for cardiovascular diseases (CVDs). Oxidative stress is a contributing factor to the pathogenesis of obesity. Meanwhile, dietary antioxidants may improve the antioxidant defense system and thereby decrease oxidative injury. Dietary total antioxidant capacity (TAC) is usually used to investigate the potential health effects of dietary antioxidant intake on several oxidative stress induced chronic diseases. This study aimed to examine the association of dietary TAC with obesity-related features in T2DM patients.

**Methods:**

The present study included 254 type 2 diabetes outpatients with a mean (SD) age of 54.52 (7.21) years and mean (SD) diabetes duration of 8.2 (6.4) years. Data on dietary intake was assessed using a validated food frequency questionnaire. Dietary TAC was estimated by ferric reducing antioxidant potential (FRAP) method. Anthropometric, clinical and lifestyle characteristics were all collected.

**Results:**

In linear regression analyses, dietary antioxidant capacity was inversely associated with body mass index (β = −0,231; 95% CI, −0,419 to −0,042), waist circumference (β = −0,427; 95% CI, −0,849 to −0,006) and fat mass percentage (β = −0,328; 95% CI, −0,545 to −0,112) independently of the assessed confounding variables. Interestingly, dietary TAC showed positive and significant associations with vitamin A, vitamin C, β-carotene, magnesium, folic acid and iron intakes, after adjusting for age and daily energy intake.

**Conclusions:**

Higher intake of dietary TAC was in association with lower indices of general and central obesity in T2DM patients. Therefore, dietary recommendations for counteracting obesity in patients with T2DM should take into account a high dietary TAC.

## Introduction

Obesity is a multifactorial and preventable disorder, which substantially increases the risk of a broad range of chronic diseases [1]. According to the World Obesity Atlas 2023, 51% of the world population will be overweight or obese by 2035. In Morocco, 46% of the adult population is at risk of being overweight or obese by 2035, according to experts from the World Obesity Federation, which alerts us to a "very high" risk [2].

Obesity and type 2 diabetes are closely interlinked. A recent systematic review and meta-analysis study showed a high prevalence of overweight and obesity among patients with type 2 diabetes in Africa [3]. The relationship between obesity and T2DM has been well-established in the scientific literature with the term “Diabesity” coined to underline their bidirectional relationship [4]. Type 2 diabetes is the major form of diabetes mellitus related to the risk of developing cardiovascular events, in which the obesity is potentially an independent risk factor [5]. A higher adiposity might induce chronic inflammation and signal pathways triggering, in turn, other metabolic disturbances [6]. Nonetheless, type 2 diabetes can increase the risk of weight gain, partly due to the excessive caloric intake by diabetes individuals to compensate for the increasing demand for energy owing to insulin resistance. In addition, chronic hyperglycemia may alter nutrient metabolism and lead to excessive free radical generation [7]. Increasing oxidative stress can be contributing factors to the development of obesity [8]. Anthropometric indices such as Body Mass Index (BMI) has been shown to be closely related to oxidative stress [9].

For type 2 diabetic patients, obesity will be a common comorbidity that may significantly worsen metabolic control and contribute to poor outcomes [10]. The prevention of weight gain and modest weight losses can reduce diabetes-related complications and significantly improve cardiovascular disease risk factors [11, 12]. For this reason, the American Diabetes Association recommends weight losses of 5% for type 2 diabetes patients with overweight or obesity in order to improve their healthcare and well-being [13].

The relationships between dietary intake and adiposity have been previously established and the intake of antioxidants from food appears to potentially elicit a beneficial effect on obesity [14–16]. In some studies, antioxidants such as vitamin A, vitamin C, selenium and zinc were inversely related to adiposity [17–19]. However, one single food item or individual antioxidant may not reflect the total antioxidant potential of the diet, since the whole diet contains a variety of antioxidants with cumulative or synergistic effects. Therefore, the total dietary antioxidant capacity has been developed to evaluate the integrated antioxidant capacity of all antioxidants present in food [20]. Dietary TAC has been usually used as an epidemiological tool to assess the dietary intake of antioxidants and their potential health benefits. In this sense, increased dietary TAC was associated with higher quality of the diet [21], improved plasma antioxidant capacity and inflammatory status [22–24]. In previous population-based studies, dietary TAC showed an inverse association with metabolic disorders and risk of CVDs [25, 26].

Our previous study documented the total dietary antioxidant capacity average and its dietary sources among Moroccan adults with Type 2 Diabetes [27]. In light of the importance of obesity in the clinic population of T2DM patients, the current study aimed to explore the relationship between dietary TAC and obesity-related features in Moroccan patients with T2DM.

## Materials and methods

### Study design and population

As previously described in our recent publication [27], this cross-sectional study was carried out in Tetouan, a city located in the extreme north of Morocco, among 254 outpatients with T2DM, aged 34–76 years old. The studied participants were recruited between the 15 February 2021 and 28 July 2022, when they attended the primary health care centers and the health department of endocrinology and metabolic diseases of the provincial hospital of Tetouan, for regular medical examination. Outpatients followed for T2DM for at least one year who voluntarily underwent anthropometric measurement, demographic data and dietary assessment were qualified for this study.

All participants completed a written informed consent form after a clear explanation of the study protocol, which was previously approved by the Ethics committee of University Hospital Center of Fez (ref. n° 06/20).

### Physical activity, anthropometric and body composition assessment

#### Physical activity

Information on physical activity was collected by a trained researcher during a face-to-face interview. To estimate the level of physical activity, the version translated to Arabic of Global Physical Activity Questionnaire (GPAQ) was used [28]. This questionnaire consists of questions in three physical activity domains: job-related activities; travel to and from places and recreation activities as well as sedentary behaviors. Data on physical activity were then analysed and metabolic equivalent hour per week (METs—h per week) values were calculated as described elsewhere [29].

Sedentary behavior was assessed through the daily number of hours of sitting position excluding the time spent in sleeping.

#### Anthropometric and body composition measurements

Assessment of body composition was conducted using impedance meter scale. Standing height without shoes was measured by wall-mounted calibrated stadiometer. The BMI was calculated as weight in kilograms divided by the square of the height in meters (kg/m2), which was used as a general obesity indicator, according to the WHO criteria [30]. Waist circumference at the umbilical level, was measured using an anthropometric measuring tape with the participant in the standing position. Waist circumference values greater than 102 cm for men and 88 cm for women were considered an indicator of abdominal adiposity [31].

Systolic and diastolic blood pressure was measured using automatic Omron tensiometer for three times and the average of the last two blood pressure measurements was considered for this analysis. Arterial hypertension was defined as a systolic blood pressure level ≥ 140 mmHg and/or diastolic blood pressure level ≥ 90 mmHg or taking antihypertensive medication [32].

### Dietary intake assessment

The usual dietary intake of type 2 diabetes patients was assessed by a trained dietitian using a validated 1-year-semi-quantitative food frequency questionnaire (S-FFQ) targeted to Moroccan population [33]. This FFQ with 255 food items has shown a good relative validity compared to the dietary average obtained from three 24-hour recalls and there were no sex differences regarding the reliability of reported dietary intakes, as reported in detail elsewhere [33].

Food consumption for each item was questioned based on a daily, weekly, or monthly frequency versus portion size. The reported frequency was then converted to the amount of each several consumed food items in grams per day. Total energy and nutrient intakes from the diet was calculated based on the amount of food consumed with the use of Tunisian and Moroccan Food Composition Tables [34, 35].

### Assessment of dietary total antioxidant capacity

To assess the dietary antioxidant capacity from the food frequency questionnaire, we used a FRAP (ferric reducing antioxidant potential) value for each dietary item, based on their ability to reduce ferric iron (Fe^3+^) to ferrous iron (Fe^2+^) using Antioxidant Food Table established by Carlson et al. [36, 37]. The current FRAP database includes more than 3100 different foods, beverages, spices, herbs, and supplements purchased from different countries. We multiplied the daily intake of foods and beverages for each participant by their corresponding antioxidant content. Then, the dietary TAC is a sum of all TAC values from each food item consumed in mmol per day.

For foods for which the antioxidant content were not available on the database, the value of the closest comparable foodstuff was used as a proxy: for bakkoula (Moroccan salad recipe based on leaves similar to spinach) the FRAP value for spinach has been used, for dchicha (traditional Moroccan soup based on barley semolina) and saykouk (a Moroccan dish made from barley couscous and buttermilk) the estimated TAC for the barley was used. For cooked foods with missing FRAP values, the value of the fresh food was considered.

### Other variable assessment

Additional information on demographic data was collected using a structured questionnaire based on the STEPS instrument as documented in our previous study [27].

### Statistical analysis

The normality of distributions was tested by the Kolmogorov–Smirnov test. Continuous variables were compared between individuals with dietary antioxidants higher and lower than the median value (cut-off value of 10.6 mmol/day) using the parametric Student’s t-test or nonparametric Mann–Whitney U test. Comparison of categorical variables was conducted using the chi-squared test.

Partial correlations adjusted by age and energy intake were used to evaluate the association between dietary TAC and daily antioxidant intake.

A multivariate linear regression analysis was performed to further elucidate the association between dietary TAC (as independent variable) and anthropometric adipose measures (as dependent variables). This model was controlled by age, physical activity and sedentary behavior. Results are expressed as mean (standard deviation) for continuous variables and frequency for categorical variables, while 95% confidence intervals are used to describe linear regression coefficient (*β)* values.

Statistical analyses were performed using SPSS version 25.0 for Windows XP. All *p*-values were two-tailed. Values of *p* < 0.05 were considered statistically significant.

## Results

The mean (SD) age of the participants was 54.52 (7.21) years old and the mean (SD) diabetes duration was 8.2 (6.4) years. Regarding the anthropometric parameters, the average value of BMI exceeded the overweight border, the mean BMI was 30.46 ± 5.16 kg/m^2^ and the mean waist circumference was 95.60 ± 11.46 cm. The frequency of overweight and obesity was 39.8% and 47.2% respectively, while abdominal obesity prevailed in 67.6% of studied diabetes outpatients.

Anthropometric data, clinical and lifestyle characteristics of participants were compared with the median value of dietary TAC (10.6 mmol day^-1^) **(Table 1).** There was significant difference between diabetes subjects with higher and lower dietary antioxidant capacity regarding age (*p* = 0.005) and diabetes duration (*p* = 0.044). The diabetes subjects with a diet high in TAC had significantly lower BMI and waist circumference values as well as lower body fat percentage, while reported higher body muscle percentage as compared with those of the lower dietary antioxidant capacity (*p* <0.05). Moreover, the diabetes patients with greater dietary antioxidant capacity had a significantly lower occurrence of obesity (as BMI ≥ 30) in comparison to those with a lower dietary TAC. Regarding the lifestyle features, those diabetes patients with a higher dietary antioxidant capacity presented higher levels of physical activity and also spent more time for leisure physical activity (*p* <0.05 for all comparisons).

**Table 1:** Anthropometric, clinical, and lifestyle characteristics of patients with T2DM according to dietary TAC.

Variables	Dietary TAC	Dietary TAC	*p- value* ^†^
	< 10.6 mmol	> 10.6 mmol	
	(n = 127)		(n = 127)	
**Age** *(years)*	55.74 ± 6.41	53.29 ± 7.75	0.005
**Gender,** n (%)			0.856
Female	109 (85.8)	110 (86.6)	
Male	18 (14.2)	17 (13.4)	
**Diabetes duration** *(years)*	7.73 ± 6.77	8.71 ± 6.01	0.044
**Family history of CVD**, yes *n (%)*	45(35.4)	43(33.9)	0.792
**Diabesity**, *n (%)*	68(53.5)	50(39.4)	0.024
**Abdominal obesity**, *n (%)*	87(69.6)	82(65.6)	0.499
**Hypertension,** *n (%)*	95(74.8)	82(64.6)	0.076
**Body weight** *(kg)*	78.79 ± 12.70	76.20 ± 13.22	0.061
**Height** *(cm)*	159.12 ± 7.46	160.28 ±7.80	0.280
**Body mass index** *(kg/m*^*2*^*)*	31.14 ± 4.70	29.77 ± 5.52	0.007
**Body mass index** *(kg/m*^*2*^*)*, *n (%)*			0.011
*Normal (< 25)*	10(7.9)	23(18.1)	
*Overweight (25–30)*	47(37)	54(42.5)	
*Obesity (≥30)*	70(55.1)	50(39.4)	
**Waist circumference** *(cm)*	96.94 ± 11.45	94.26 ± 11.36	0.028
**Body fat** (%)	37.14 ± 5.14	35.19 ± 6.51	0.017
**Body water** (%)	46.34 ± 3.84	47.70 ± 5.06	0.017
**Body muscle** (%)	30.19 ± 3.19	30.88 ± 3.63	0.009
**Systolic Blood pressure** *(mmHg)*	142.78 ± 20.09	138.73 ± 19.81	0.108
**Diastolic Blood pressure** *(mmHg*)	80.79 ± 11.84	80.26 ± 10.62	0.760
**Total physical activity**. *METs-h per week*	42.45 ± 38.27	58.52 ± 52.80	0.006
**Leisure-time physical activity**. *METs-h per week*	6.96 ± 12.97	8.96 ± 13.03	0.023
**Sedentary behavior**. *h per day*	9.43 ± 2.47	9.35 ± 2.58	0.795

Dietary TAC was categorized as low (<10.6 mmol, n = 127) and high (>10.6 mmol, n = 127) values, according to the median value (10.6 mmol). Continuous data are expressed as mean ± standard deviation and frequency for categorical variables.

† Obtained from student t-test or the Mann-Whitney U test for continuous variables and chi-square test for categorical variables. Differences were considered when *p* < 0.05.

CVD, cardiovascular disease; MET, metabolic equivalent of task; mmHg millimeters of mercury

The dietary characteristics of participants were compared according to the median value of dietary antioxidant capacity (10.6 mmol day-1) **(Table 2).** In comparison with the diabetes who had lower dietary antioxidant capacity values than the median, those with higher dietary TAC had higher intakes of total energy, protein, carbohydrate, total fat, fiber and micronutrients with antioxidant effects as well as higher consumption of almost all food groups.

**Table 2:** Nutrients and food intakes of T2DM patients according to dietary TAC.

	Dietary TAC	Dietary TAC	*p- value* ^*†*^
	< 10.6 mmol	> 10.6 mmol	
	(n = 127)	(n = 127)	
Total energy (kcal/day)	2257.0 ± 524.2	2866.9 ± 608.1	< 0.001
Carbohydrate (g/day)	287.6 ± 79.8	360.4 ± 88.6	< 0.001
Protein (g/day)	79.4 ± 20.8	99.0 ± 24.3	< 0.001
Total fat (g/day)	99.6 ± 35.4	129.0 ± 37.2	< 0.001
Fiber (g/day)	44.0 ± 15.7	58.5 ± 21.0	< 0.001
Folic acid (mcg/day)	896.7 ± 375.8	1267.6 ± 457.9	< 0.001
Vitamin A (mcg/day)	471.6 ± 225.8	613.1 ± 279.6	< 0.001
Vitamin E (mg/day)	18.8 ± 7.1	24.7± 7.5	< 0.001
Vitamin C (mg/day)	81.0 ± 36.6	118.7 ± 51.9	< 0.001
Vitamin D (mcg/day)	6.2± 3.2	7.3 ± 3.3	0.007
β-carotene (mcg/day)	3320.2 ± 2252.9	4309.7 ± 2881.2	0.006
Magnesium (mg/day)	538.1 ± 165.5	765.9 ± 233.3	< 0.001
Iron (mg/day)	48.6 ± 42.1	74.7 ± 59.7	< 0.001
Zinc (mg/day)	24.8 ± 16.7	35.4 ± 18.1	< 0.001
Selenium (mcg/day)	123.2 ± 33.0	144.4 ± 33.1	< 0.001
**Foods (g/day)**			
Vegetables	237.6 ± 113.9	381.7 ± 155.1	< 0.001
Potatoes	66.2 ± 51.0	77.2 ± 51.9	0.018
Legumes	117.8 ± 95.9	148.2 ± 108.3	0.016
Wholegrain cereal	177.0 ± 162.0	212.2 ± 167.8	0.038
Refined grain cereal	187.7 ± 154.8	209.7 ± 161.4	0.207
Fruits	170.1 ± 97.7	259.4 ± 140.2	< 0.001
Meat	14.55 ±16.90	17.16 ±19.21	0.124
Poultry	28.21 ± 21.86	31.95 ± 21.06	0.078
Eggs	15.03 ± 11.95	20.73 ± 22.78	0.059
Fish	43.9 ± 25.5	54.1 ± 26.3	0.002
Dairy products	83.40 ±76.13	114.74 ± 110.76	0.013
Tea infusion	120.4 ± 101.6	236.0 ± 173.6	< 0.001
Coffee infusion	68.8 ± 64.4	117.37 ± 117.36	0.002
Nuts and seeds	1.8 ± 4.6	5.0 ± 9.8	0.004
Olives Oil	27.8 ± 19.8	37.3 ± 20.3	< 0.001

Dietary TAC was categorized as low (<10.6 mmol, n = 127) and high (>10.6 mmol, n = 127) values, according to the median value (10.6 mmol). All values are mean ± SD. TAC total antioxidant capacity. Kcal kilocalorie, g gram, mg milligram, mcg microgram

^**†**^ *p -*value is for student t test or Mann-Whitney U test.

Partial correlations were performed between TAC values of the diet and some antioxidant intakes **(Table 3).** After adjusting for age and daily energy intake, folic acid, β-carotene, vitamins A and C, magnesium and iron showed a positive and significant correlation with dietary antioxidant capacity.

**Table 3:** Dietary TAC as a measurement of antioxidant intake †.

Antioxidant daily intake	*Dietary Total Antioxidant Capacity (mmol/Day)*	
	r	*p- value*
Folic acid (mcg/day)	0.246	< 0.001
Vitamin A (mcg/day)	0.126	0.046
Vitamin C (mg/day)	0.325	< 0.001
β-carotene (mcg/day)	0.125	0.048
Magnesium (mg / day)	0.346	< 0.001
Iron (mg/day)	0.169	0.007

† Partial correlations between dietary TAC value and antioxidant daily intake of the subjects were adjusted by age and daily energy intake.

To further investigate the relationships between obesity indices and dietary antioxidant capacity value, a multivariate linear regression analysis was developed **(Table 4).** The BMI, waist circumference and total body fat average appeared to be inversely associated with dietary antioxidant capacity independent of age, physical activity and sedentary behaviors. As regards the TAC coefficient values, waist circumference was the adiposity index with a higher association with dietary antioxidant capacity.

**Table 4:** Association between dietary TAC and anthropometric adipose measures.

	Model 1			Model 2		
Anthropometric variables	β (95% CI)	*p*	R^2^	β (95% CI)	*p*	R^2^
**Body mass index** (kg/m^2^)	−0.184 (−0,370 to 0,002)	0.052	0.01	−0,231 (−0,419 to −0,042)	0.017	0.05
**Waist circumference** (cm)	−0,362 (−0,779 to 0,055)	0.088	0.01	−0,427 (−0,849 to −0,006)	0.047	0.05
**Total body fat** (%)	−0,302 (−0,514 to −0,090)	0,005	0.03	−0,328 (−0,545 to −0,112)	0,003	0.05

Results are presented as β (regression coefficient) and 95% CI (confidence interval); Model 1: crude analysis, Model 2: multivariate linear regression adjusted for age, physical activity METs-h per week and sedentary behavior h per day.

## Discussion

In the present study, we evaluated for the first time the relationship between dietary TAC, measured by the FRAP method, and obesity indices among Moroccan type 2 diabetes patients. It was found that higher dietary TAC was associated with a lower average BMI, waist circumference and total body fat. This association was independent of potential confounders.

As in this work, negative association between BMI and dietary antioxidant capacity has been reported in a cross-sectional study among chronic kidney disease patients [38]. In addition, there was a significant inverse correlation between dietary TAC and waist circumference as well as total body fat percentage. In another cross-sectional study conducted by Hermsdorff et al. [39], participants in the highest tertile of dietary TAC had 4 cm lower measure of waist circumference and they showed lower abdominal adiposity than those in the lowest tertile. Furthermore, there was a significant negative partial correlation between dietary TAC and waist circumference. Interestingly, being overweight was found associated with low intake of dietary antioxidants in a cross-sectional designed study of healthy subjects aged 25–50 years old [40]. A cohort study by Bahadoran et al. [41] performed in the healthy adults from the Tehran Lipid and Glucose Study, indicated that waist circumference was significantly lower in the highest versus the lowest quartile of dietary TAC. Decreased occurrence of abdominal obesity across quartiles of dietary TAC has been also reported after three years of follow-up. In another cohort study conducted in middle-aged and elderly participants of the Rotterdam Study, a higher FRAP score was associated with a lower body fat percentage [42]. Findings from the RESMENA (MEtabolic Syndrome REduction in NAvarra-Spain) randomized controlled trial [43] indicated that total dietary antioxidant capacity showed the highest contribution to reduced body mass and obesity indices in adults with metabolic syndrome features. A recent systematic review and meta-analysis study [44] reported a significant association between higher dietary TAC and reduced prevalence of central obesity and waist circumference measurement. The present study as the aforementioned studies support previous research that highlights the inverse relationship between plasma antioxidant capacity, regarded as a representative indicator of dietary TAC, and obesity indices [45].

The dietary TAC, in addition to measuring antioxidant intake, can be considered an indicator of healthy diet quality [26], allowing a comparison between our findings and studies that assessed diet quality using well-known dietary indices. In a study conducted by Mohtadi et al. [46], the overweight in Moroccan adults was inversely associated with the quality of the diet, evaluated using the simplified Mediterranean-Diet score (MD score). In another cross-sectional study among Moroccan adults, being obese was one of the factors associated with poor adherence to a Mediterranean diet [47]. Findings from a meta-analysis study [48] have shown that reduced waist circumference was associated with a higher adherence to the Mediterranean pattern. In addition, Asghari et al. [49] reported an inverse association between obesity and diet quality as assessed by the Healthy Eating Index (HEI). In obese individuals, a higher BMI and body fat were associated to a Western Diet [50].

As regards the distribution of descriptive features of patients with T2DM by dietary TAC, the finding that diabetic patients with low physical activity behavior and high occurrence of obesity consumed a diet with a lower TAC can be a serious health concern. The lack of physical activity and increased body fat are known common risk factors associated with CVDs in diabetes patients. Then, in these specific conditions, a greater dietary TAC intake is recommended, since it appears to be beneficially associated with cardiometabolic risk factors [51].

As expected, our findings indicated that a higher dietary TAC was associated with a higher consumption of vegetables, fruits, coffee, tea, and nuts, which have been also reported previously [52].

In this study, diabetes patients with higher dietary TAC compared to those with lower dietary antioxidant capacity had a greater intake of dietary fibre and higher consumption of plant-based foods such as whole grains, vegetables, fruits, nuts and legumes. According to a recent meta-analysis of prospective studies, food groups associated with reduced risk of overweight, obesity and weight gain include whole grains, fruits, legumes and nuts [53]. The metabolic health benefits of dietary fiber in type 2 diabetes have also long been established. A high-fiber diet might contribute to reduced risk of obesity, as substantiated by its potential effects on decreasing absorption of macronutrients in the gastrointestinal tract, increasing satiety, improving the gut microbiota and therefore reducing the risk of gaining weight [54].

Most studies have demonstrated that increased serum TAC was associated with increased dietary TAC [55, 56], suggesting that dietary TAC may be a useful tool to assess the antioxidant intake. In our study, dietary FRAP was positively associated with the intake of individual dietary antioxidants, such as vitamin C, vitamin A, β-carotene, iron and magnesium. This finding is consistent with previous studies investigating dietary TAC as a measurement of antioxidant intakes [57, 58].

Some limitations of the current study should be underlined. First, the study had a cross- sectional design; so, the relationship between dietary TAC and obesity-related features of Moroccan type 2 diabetes patients could not be inferred as a causal relation. Future studies are needed to establish causality. Second, antioxidant parameters in plasma of diabetes participants were not examined. Nevertheless, previous clinical studies investigated the plasmatic antioxidant status in Moroccan patients with type 2 diabetes [59]. Third, the dietary TAC assessment was based on an international database, in which the values may vary in relation to foods produced in Morocco. Therefore, we selected the largest database that reported total antioxidant content of food items consumed worldwide and which covered most of the foods eaten in Morocco. A strong point of the current study includes its originality. As far as we know, this is the first study examining the relationship between dietary TAC and obesity indices among Moroccan type 2 diabetes patients. Furthermore, the data collection was performed by a qualified nutritionist, and the questionnaire used, even though is prone to measurement error, it was detailed and validated for use in the Moroccan adult population, hence capturing most of the antioxidant-rich food items.

## Conclusion

As evidenced throughout this study, there is an inverse relationship between dietary total antioxidant capacity, measured by the FRAP method, and obesity-related features in patients with T2DM, suggesting that a dietary pattern with a high-antioxidant potential is related to a healthier diet and can help control the excess weight gain and obesity, particularly abdominal obesity in this clinical population. Therefore, dietary recommendations for tackling obesity in T2DM patients should take into account a high dietary TAC.
